# Serum S100A8/A9 concentrations are associated with neuropsychiatric involvement in systemic lupus erythematosus: a cross-sectional study

**DOI:** 10.1186/s41927-022-00268-w

**Published:** 2022-07-09

**Authors:** Kristoffer A. Zervides, Andreas Jern, Jessika Nystedt, Birgitta Gullstrand, Petra C. Nilsson, Pia C. Sundgren, Anders A. Bengtsson, Andreas Jönsen

**Affiliations:** 1 Department of Clinical Sciences, Rheumatology, Lund University, Skåne University Hospital, Lund, Sweden; 2 Department of Clinical Sciences, Neurology, Lund University, Skåne University Hospital, Lund, Sweden; 3 Department of Clinical Sciences, Diagnostic Radiology, Lund University, Skåne University Hospital, Lund, Sweden; 4 Lund University BioImaging Center, Lund University, Lund, Sweden

**Keywords:** Systemic lupus erythematosus, Neuropsychiatric systemic lupus erythematosus, S100A8/A9, Fatigue, Inflammation, Serum, Cerebrospinal fluid, Biomarker

## Abstract

**Background:**

Neuropsychiatric (NP) involvement and fatigue are major problems in systemic lupus erythematosus (SLE). S100A8/A9 is a marker of inflammation and responds to therapy in SLE patients. S100A8/A9 has an immunopathogenic role in various neurological diseases. We investigated S100A8/A9 in relation to NP-involvement and fatigue in SLE.

**Methods:**

72 consecutive SLE outpatients at a tertiary centre and 26 healthy controls were included in this cross-sectional study. NPSLE was determined by specialists in rheumatology and neurology and defined according to three attribution models: “ACR”, “SLICC A” and “SLICC B”. Cerebral MRI was assessed by a neuroradiologist and neurocognitive testing by a neuropsychologist. The individuals were assessed by scores of pain (VAS), fatigue (VAS and FSS), and depression (MADRS-S). Concentrations of S100A8/A9 in serum and cerebrospinal fluid were measured with ELISA. Statistical calculations were performed using non-parametric methods.

**Results:**

Serum concentrations of S100A8/A9 were higher in SLE patients compared with controls (medians 1230 ng/ml; 790 ng/ml, *p* = 0.023). The concentrations were higher in NPSLE patients compared with non-NPSLE patients when applying the SLICC A and ACR models, but not significant when applying the SLICC B model (medians 1400 ng/ml; 920 ng/ml, *p* = 0.011; 1560 ng/ml; 1090 ng/ml, *p* = 0.050; 1460 ng/ml; 1090 ng/ml, *p* = 0.083, respectively). No differences of CSF S100A8/A9 concentrations were observed between NPSLE and non-NPSLE patients. SLE patients with depression or cognitive dysfunction as an ACR NPSLE manifestation had higher serum S100A8/A9 concentrations than non-NPSLE patients (median 1460 ng/ml, *p* = 0.007 and 1380 ng/ml, *p* = 0.013, respectively). Higher serum S100A8/A9 correlated with higher VAS fatigue (r = 0.31; *p* = 0.008) and VAS pain (r = 0.27, *p* = 0.021) in SLE patients. Serum S100A8/A9 was not independently associated with NPSLE when adjusting for scores of fatigue (FSS) and pain (VAS) (OR 1.86, 95% CI 0.93–3.73, *p* = 0.08).

**Conclusions:**

Serum S100A8/A9 concentrations may be associated with NPSLE and fatigue. S100A8/A9 may be of interest in evaluating NPSLE, although further investigations are needed.

## Background

Systemic lupus erythematosus (SLE) is a relapsing–remitting chronic autoimmune inflammatory disease which can affect several organs, such as the skin, joints, kidneys, and nervous system. SLE mostly affects women (85%) and is on average diagnosed before the age of 50 [1]. Neuropsychiatric (NP) involvement is estimated to affect 12–95% of SLE-patients depending on the criteria used, and is associated with poorer prognosis, reduced quality of life, and lower work capability [2, 3]. The NP symptoms vary from severe central nervous system (CNS) manifestations, such as stroke, seizures, myelopathy and psychosis, to more common manifestations such as cognitive disturbances, affective disorders and headache [4, 5]. The pathophysiology of NPSLE is heterogeneous and not fully understood. It includes inflammation-mediated tissue injury and ischemic vessel injury with abnormalities of the blood–brain barrier and autoantibody-mediated production of pro-inflammatory cytokines [6]. One potential manifestation of SLE is fatigue, which remains one of the most salient, poorly understood and addressed patient complaints, and has been designated the most disabling symptom of a majority of SLE patients, often severely impacting quality of life and work capability [7, 8]. Understanding the mechanisms of fatigue can help guide the interventions to improve health outcomes in SLE. The aetiology of fatigue in SLE is multifactorial, however, peripheral inflammation may cause fatigue directly by various neuroimmune pathways including microglial activation and toll-like receptor 4 (TLR4) upregulation [7, 9]. The inflammatory biomarker S100A8/A9 may have a role in NPSLE and fatigue immunopathology and will be investigated in this paper.

S100A8 and S100A9 are calgranulins, Ca^2+^-binding proteins, members of the S100 family, and often exist in an active heterodimeric complex form named S100A8/A9, MRP8/14, or calprotectin. S100A8/A9 is abundantly expressed by phagocytes and acts as an endogenous damage-associated molecular pattern. During inflammation, S100A8/A9 is released passively and actively and exerts a critical role in modulating the inflammatory response by interacting with various membrane receptors, including TLR4 and Receptor of Advanced Glycation Endproducts (RAGE), resulting in leukocyte recruitment and cytokine secretion via the NFκB-pathway [10, 11]. S100A8/A9 has been linked to the pathology of arteriosclerosis, and of various chronic inflammatory diseases such as SLE, rheumatoid arthritis, and inflammatory bowel disease [12–14]. SLE patients have higher serum S100A8/A9 levels compared with healthy controls, even when their disease is clinically inactive, and serum S100A8/A9 concentrations have been correlated with disease activity and decrease after immunosuppressive treatment [13–17]. S100A8/A9 concentrations are higher in the serum in multiple sclerosis (MS) patients [18] and in the cerebrospinal fluid (CSF) of Alzheimer’s disease (AD) patients [19] compared with healthy controls. Studies have revealed a neuroinflammatory role of S100A8/A9 in various neurological and psychiatric disorders such as AD [20, 21], Parkinson’s disease [22], depression [23], anxiety [24], MS [18], traumatic brain injury [12], and stroke [25]. The underlying mechanisms of S100A8/A9 in CNS pathology remain unclear, however, treatment with S100A8/A9 in vitro induces the activation, proliferation and migration of microglia in mice to switch from an anti-inflammatory M2 phenotype to a pro-inflammatory activated M1 phenotype, activating the NFκB-pathway leading to the production of pro-inflammatory cytokines and chemokines, consequently resulting in injury of cells in the CNS [26].

The associations between S100A8/A9 and SLE, and the fact that S100A8/A9 is expressed in the CNS and has been associated with neurological disorders, prompted us to investigate the possible association between NPSLE and S100A8/A9.

## Methods

### Patients and data collection

SLE out-patients attending the Department of Rheumatology in Lund, Skåne University Hospital, Sweden during 2011–2014 were consecutively asked to participate in this cross-sectional study. The patients were recruited independently of disease activity or NP symptoms. To reduce study group heterogeneity to facilitate interpretation of results, only female patients and patients below an age threshold of 55 were asked to participate. The latter specifically to reduce age-related cognitive decline and MRI abnormalities. Patients with any contraindication to magnetic resonance imaging (MRI) or pregnancy were not asked to participate in the study. Twenty-six age-matched healthy female controls were recruited from health care personnel from the departments of Rheumatology, Radiology and Neurosurgery.

A specialist in rheumatology and a specialist in neurology evaluated all 72 patients. NP symptoms were assessed and attributed to either SLE or other causes and required consensus between the rheumatologist and neurologist. Individual NPSLE manifestations were defined using the three attribution models. Patients with any NPSLE manifestation due to SLE defined in the American College of Rheumatology (ACR) case definitions are herein classified as NPSLE according to the ACR model [27]. In addition, patients were classified according to the more stringent NPSLE attribution models “Systemic Lupus Erythematosus International Collaborating Clinics (SLICC) A” and “SLICC B” defined by Hanly et al. [3]. In the most stringent model, SLICC A, all NP events more than 6 months prior to SLE diagnosis are not considered to be caused by SLE. In SLICC B, all NP events more than 10 years prior to SLE diagnosis are not considered to be caused by SLE. In both SLICC A and B, non-SLE factors responsible for an NP event, and all minor NP events are not considered to be caused by SLE. Minor NP events are defined by Ainiala et al. as events found to be as common in the population as among SLE patients, namely mild cognitive dysfunction, mild depression, anxiety, headache and neuropathy without objective findings [5, 28].

Organ damage was recorded according to the SLICC/ACR-Damage Index (SLICC/ACR-DI) [29]. SLE disease activity was assessed using the SLE Disease Activity Index 2000 (SLEDAI-2 K) [30]. All patients fulfilled the SLICC classification criteria for SLE [31]. Measurements of fatigue were recorded using the Fatigue Severity Scale (FSS) (sum of score 9–63) and the Visual Analogue Scale 100 mm (VAS). For FSS, fatigue was defined clinically significant with FSS total ≥ 36, with moderate fatigue between 36 and 52, and severe fatigue more than 52 [32]. Pain was recorded using the VAS. For the VAS questions, the patients were asked to assess their fatigue and their pain caused by their SLE during the last week. Depression was evaluated by the Montgomery-Åsberg Depression Rating Scale – Self-rated version (MADRS-S). Cognitive dysfunction was determined by using the CNS Vital Signs Battery (CNS-VS), a computerized neurocognitive test battery consisting of seven cognitive tests by a neuropsychologist, producing age-adjusted scores in twelve BRIEF-CORE Clinical Domanis [33]. Cerebral MR Imaging was performed on a MAGNETOM Skyra 3 T system (Siemens Healthcare, Erlangen, Germany), and the data was evaluated by a neuroradiologist for alterations in brain volumes and white matter abnormalities as described in a previous study [34].

All patients without contraindications were invited to undergo lumbar puncture. In all, 33 patients accepted the procedure (12 non-NPSLE patients and 21 NPSLE patients according to the ACR model). CSF-measurements included isoelectric focusing of IgG, albumin index and concentrations of leukocytes and erythrocytes**.**

Serum and plasma samples were obtained from all patients and controls. The controls also underwent MRI and completed questionnaires, notably excluding VAS fatigue and VAS pain because these ask the patient to assess fatigue and pain due to a specific illness. Plasma, serum and CSF were stored in − 80 degrees Celsius. The concentrations of S100A8/A9 in CSF and serum were measured with the Bühlmann MRP8/14 ELISA kit, Switzerland, according to the manufacturers’ instructions [14]. The concentrations of S100A8/A9 in CSF were below the detection limit of 20 ng/ml in this kit, instead we used the ThermoFisher Human Calprotectin L1/S100-A8/A9 Complex ELISA Kit (EH62RB) (standard curve range 32.77–8000 pg/ml) for the CSF analyses. Routine biochemical and immunological analyses were performed at the Departments of Laboratory Medicine and Immunology, Skåne University Hospital, including measurements of serum levels of complement factors, anti-double-stranded DNA antibodies (anti-dsDNA), and antiphospholipid antibodies (aPL, including serum IgG anti-cardiolipin antibodies, serum IgG anti-beta-2-glycoprotein-1 antibodies) and Lupus Anticoagulant). All methods were carried out in accordance with relevant guidelines and regulations.

### Statistics

The distribution of data between groups were assessed with the Mann–Whitney *U* test. Spearman’s Rank Correlation test was used to compare continuous variables. Chi-Square, or Fisher’s Exact Test for small samples, was applied for comparing categorical data. Binary Logistic Regression was used for the multivariate analysis to understand the association between NPSLE (the binary dependent) and serum S100A8/A9 corrected for possible confounders (the continuous covariates). To avoid multicollinearity, covariates with a mutual correlation coefficient above 0.4 were not included in the multivariate model. All p-values were considered significant at p < 0.05. Since the study is considered exploratory, correction for multiple testing was not performed. IBM SPSS Statistics version 25 was used for all statistical analyses.

## Results

### Clinical characteristics

An overview of the clinical characteristics of the 72 SLE patients is presented in Table 1. Most patients had ongoing immunosuppression such as prednisolone (79%), hydroxychloroquine (79%), or any other disease-modifying anti-rheumatic drug (DMARD) (60%). SLE disease activity (SLEDAI-2 K median 2, range 0–18) and organ damage (SLICC/ACR-DI median 0, range 0–5) were low in general. The median disease duration was 10 years (range 0–32), and the median age was 38 years (range 18–52). The age of controls did not differ from the SLE group (median (range) 40 (23–52), *p* = 0.38). The majority of the SLE patients had clinically significant fatigue according to the FSS: 46% had moderate fatigue and 23% had severe fatigue, as opposed to 4% and 0% of the healthy controls.

**Table 1:** Clinical characteristics and ongoing treatment of 72 SLE patients

Clinical characteristics	
Age at study, median (range), years	38 (18–52)
Disease duration, median (range), years	10 (0–32)
SLICC/ACR-Damage Index, median (range)	0 (0–5)
SLEDAI-2 K, median (range)	2 (0–18)
*SLICC classification criteria*	
Acute cutaneous lupus, n (%)	53 (74%)
Chronic cutaneous lupus, n (%)	18 (25%)
Oral or nasal ulcers, n (%)	31 (43%)
Nonscarring alopecia, n (%)	24 (33%)
Joint disease, n (%)	62 (86%)
Serositis, n (%)	29 (40%)
Renal manifestations, n (%)	29 (40%)
Neurologic manifestations, n (%)	13 (18%)
Haemolytic anaemia, n (%)	4 (6%)
Leukopenia/lymphopenia, n (%)	42 (58%)
Thrombocytopenia, n (%)	20 (28%)
ANA, n (%)	71 (99%)
Anti-dsDNA, n (%)	44 (61%)
Anti-Sm, n (%)	11 (15%)
Anti-PL, n (%)	24 (33%)
Low complement, n (%)	43 (60%)
Positive Direct Coombs test, n (%)	2 (3%)
*Medication, ongoing*	
Cyclophosphamide, n (%)	1 (1%)
Azathioprine, n (%)	23 (32%)
Mycophenolate, n (%)	16 (22%)
Rituximab, n (%)	1 (1%)
Methotrexate, n (%)	1 (1%)
Belimumab, n (%)	8 (11%)
Hydroxychloroquine, n (%)	57 (79%)
Any DMARD except hydroxychloroquine, n (%)	43 (60%)
Intravenous immunoglobulin, n (%)	2 (3%)
Prednisolone, n (%)	57 (79%)
Prednisolone daily dose, median (range), mg/day	5 (0–25)

SLICC: Systemic lupus erythematosus international collaborating clinics. ACR: american college of rheumatology. SLEDAI-2 K: SLE disease activity Index 2000. ANA: Antinuclear antibody. Anti-dsDNA: anti-double stranded DNA. Anti-Sm: anti-smith. Anti-PL: anti-phospholipid antibody. DMARD: disease-modifying antirheumatic drug

An overview of the NPSLE manifestations is presented in Table 2. When applying the SLICC A and SLICC B models 16 (22%) and 23 (32%) SLE patients, respectively, had NPSLE. The most frequent NPSLE-manifestations were autonomic neuropathy and cranial neuropathy in both SLICC models. Forty-four (61%) SLE patients had NPSLE according to the ACR model. The most frequent NPSLE-manifestations were cognitive dysfunction (36%), headache (31%), depression (18%), anxiety (17%), and autonomic neuropathy (14%) in this model. In all three NPSLE attribution models, NPSLE patients did not differ significantly from non-NPSLE patients regarding their disease activity, disease duration, or age (Table 3) or regarding levels of complement factors and anti-dsDNA (data not shown). NPSLE patients according to SLICC A and B had a higher degree of SLE-related organ damage than non-NPSLE patients (Table 3).

**Table 2:** Overview of NPSLE-manifestations in 72 SLE patients when applying three NPSLE attribution models

NPSLE-manifestation	SLICC A model	SLICC B model	ACR model
Any NPSLE-manifestation, n (%)	16 (22%)	23 (32%)	44 (61%)
Cognitive dysfunction, n (%)	0 (0%)	5 (7%)	26 (36%)
Headache, n (%)	N/A	N/A	22 (31%)
Depression, n (%)	0 (0%)	1 (1%)	13 (18%)
Anxiety disorder, n (%)	N/A	N/A	12 (17%)
Autonomic neuropathy, n (%)	7 (10%)	8 (11%)	10 (14%)
Cranial neuropathy, n (%)	7 (10%)	7 (10%)	7 (10%)
Cerebrovascular disease, n (%)	1 (1%)	5 (7%)	5 (7%)
Demyelinating disease, n (%)	3 (4%)	3 (4%)	3 (4%)
Myelitis, n (%)	2 (3%)	3 (4%)	3 (4%)
Confusion, n (%)	1 (1%)	2 (3%)	3 (4%)
Polyneuropathy, n (%)	1 (1%)	1 (1%)	3 (4%)
Seizures, n (%)	1 (1%)	2 (3%)	2 (3%)
Mononeuritis, n (%)	1 (1%)	2 (3%)	2 (3%)
Aseptic meningitis, n (%)	1 (1%)	1 (1%)	1 (1%)
Psychosis, n (%)	1 (1%)	1 (1%)	1 (1%)
Chorea, n (%)	0 (0%)	1 (1%)	1 (1%)
Guillain-Barré syndrome, n (%)	0 (0%)	0 (0%)	0 (0%)
Plexopathy, n (%)	0 (0%)	0 (0%)	0 (0%)
Myasthenia gravis, n (%)	0 (0%)	0 (0%)	0 (0%)

One patient may have more than one NPSLE-manifestation. NPSLE: Neuropsychiatric Systemic Lupus Erythematosus. SLICC: Systemic Lupus Erythematosus International Collaborating Clinics. ACR: American College of Rheumatology. N/A: Not applicable

**Table 3:** S100A8/A9 concentrations and clinical characteristics in NPSLE and non-NPSLE patients applying three NPSLE attribution models

	SLICC A model			SLICC B model			ACR model		
	NPSLE	Non-NPSLE	*p *value	NPSLE	Non-NPSLE	*p *value	NPSLE	Non-NPSLE	*p *value
Serum S100A8/A9, ng/ml, median (range)	1560 (280–3520)	1090 (50–3540)	0.050	1460 (260–3520)	1090 (50–3540)	0.084	1400 (140–3540)	920 (50–3500)	0.011
CSF S100A8/A9, pg/ml, median (range)	316 (< 35–854)	351 (< 35–2045)	0.83	316 (< 35–1703)	351 (< 35–2045)	1.0	319 (< 35–1703)	416 (< 35–2045)	0.43
Age at study, median (range), years	41 (24–48)	36 (18–52)	0.23	39 (23–48)	37 (18–52)	0.90	40 (18–50)	35 (19–52)	0.28
Disease duration, median (range), years	10.5 (1–24)	9.5 (0–32)	0.18	10 (1–29)	10 (0–32)	0.90	10 (0–32)	10 (0–25)	0.43
SLICC/ACR-DI, median (range)	1 (0–5)	0 (0–4)	0.008	1 (0–5)	0 (0–4)	0.003	0 (0–5)	0 (0–3)	0.18
SLEDAI-2 K, median (range)	2 (0–12)	2 (0–18)	0.63	2 (0–12)	2 (0–18)	0.70	2 (0–18)	2 (0–12)	0.65

SLICC: Systemic Lupus Erythematosus International Collaborating Clinics. ACR: American College of Rheumatology. CSF: cerebrospinal fluid. DI: Damage Index. SLEDAI-2 K: SLE Disease Activity Index 2000

### S100A8/A9 and NPSLE

**Fig. 1:**
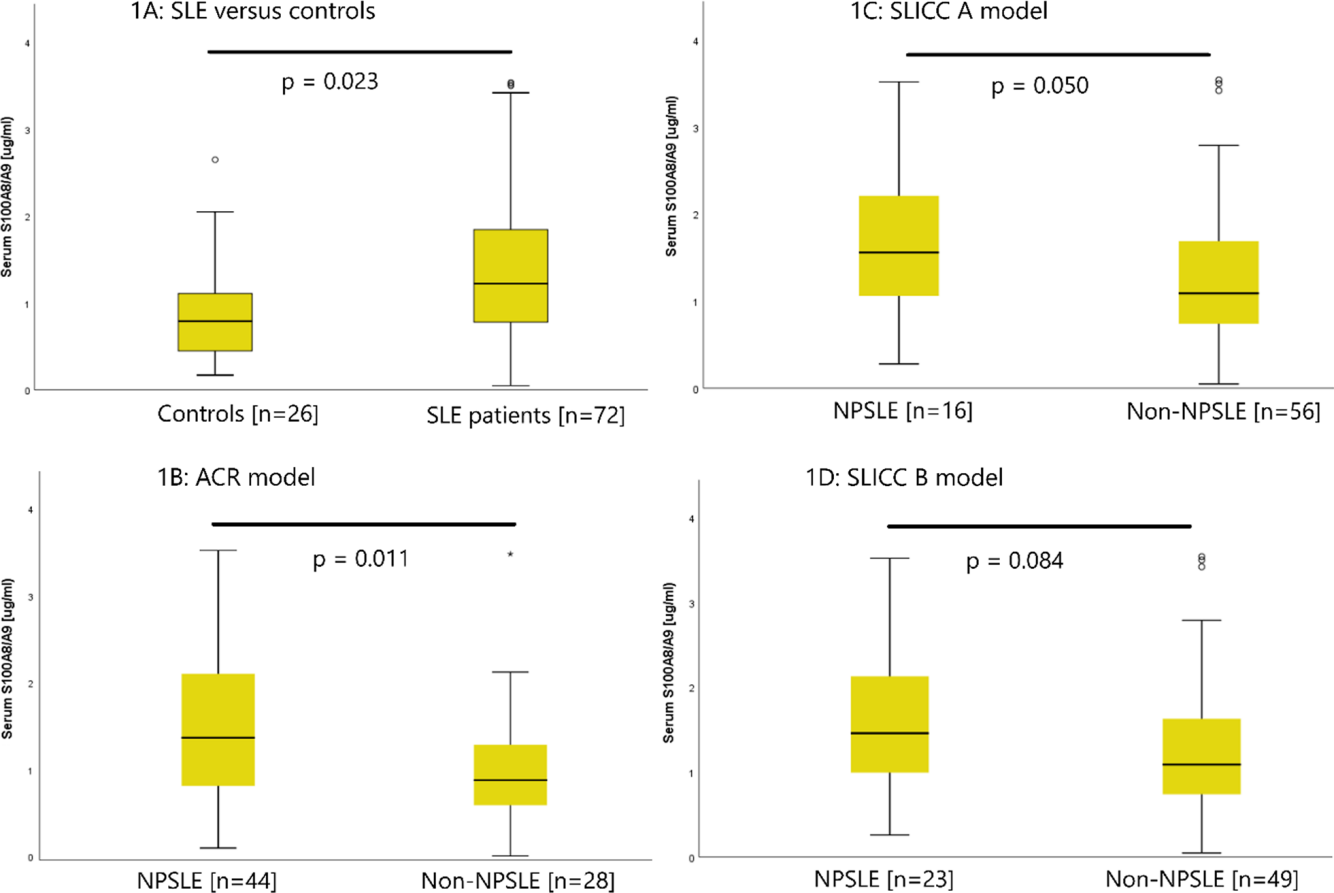
Serum S100A8/A9 concentrations between groups. The boxplots illustrate medians, quartalies and ranges of serum S100A8/A9 concentrations between groups [µg/ml]. **a** SLE patients versus healthy controls. **b**–**d** NPSLE patients versus non-NPSLE patients according to three NPSLE attribution models (**b** ACR model, **c** SLICC A model, **d** SLICC B model)

Serum S100A8/A9 concentrations were higher in SLE patients compared with healthy controls (Table 3, Fig. 1a). Serum S100A8/A9 concentrations were higher in NPSLE patients compared with non-NPSLE patients when applying the SLICC A model and the ACR model, but not significant when applying the SLICC B model (Table 3, Fig. 1b-d). Individual NPSLE manifestations according to the SLICC A and B models were not associated with higher serum S100A8/A9 concentrations (data not shown). However, SLE patients with the NPSLE manifestations “depression” or “cognitive dysfunction” had higher serum S100A8/A9 concentrations than non-NPSLE patients when applying the ACR model (Table 4).

**Table 4:** Comparison of S100A8/A9 concentrations between patients with individual NPSLE-manifestations and non-NPSLE-patients according to the NPSLE ACR model

Patient group (ACR model)	Serum S100A8/A9, ng/ml, median (range)	CSF S100A8/A9, pg/ml, median (range)
Non-NPSLE patients (reference group),	920 (50–3500)	416 (< 35–2045)
n (%)	28 (39%)	11 (34%)
Cognitive dysfunction,	1380 (260–3540)	319 (< 35–1703)
n (%)	26 (36%)	15 (47%)
*p *value	0.013	0.36
Headache,	1300 (140–2860)	252 (< 35–854)
n (%)	22 (31%)	9 (28%)
*p *value	0.058	0.37
Depression,	1460 (140–2790)	177 (< 35–526)
n (%)	13 (18%)	6 (19%)
*p *value	0.007	0.2
Anxiety,	1220 (260–2790)	143 (< 35–692)
n (%)	12 (17%)	6 (19%)
p-value	0.14	0.17
Autonomous neuropathy,	1280 (280–2440)	304 (< 35–854)
n (%)	10 (14%)	6 (19%)
*p *value	0.14	0.61
Cranial neuropathy,	1460 (280–2190)	756 (692–854)
n (%)	7 (10%)	3 (9%)
*p *value	0.087	**-**
Cerebrovascular disease,	1730 (740–2860)	692
n (%)	5 (7%)	1 (3%)
*p *value	0.1	-

Groups with less than five manifestations were not included in the analysis. P-values are for the comparison between specific NPSLE manifestations and the non-NPSLE patient group according to the ACR model. ACR: American College of Rheumatology. CSF: cerebrospinal fluid. n: number of events. %: prevalence

S100A8/A9 was detectable in the CSF of SLE patients (median (range) 351 (< 35–2045) pg/ml). No differences in CSF S100A8/A9 concentrations were observed between the non-NPSLE and NPSLE patients, regardless of attribution model, or when analysing individual NPSLE-manifestations (Tables 3 and 4).

### S100A8/A9 and correlations with clinical parameters

The correlations between serum S100A8/A9 concentrations in SLE patients and clinical parameters are depicted in Table 5. Higher serum S100A8/A9 concentrations correlated with higher VAS pain (r_s_ = 0.27, *p* = 0.021) and higher VAS fatigue (r_s_ = 0.31, *p* = 0.008) in SLE patients. Higher serum S100A8/A9 correlated with higher FSS in all individuals (r_s_ = 0.24, *p* = 0.018), although not within the SLE group (r_s_ = 0.18, *p* = 0.12).

**Table 5:** Correlations between serum S100A8/A9 and clinical parameters in 72 SLE patients

Variable	r_s_	*p *value
Age at study for SLE patient	0.054	0.65
Disease duration	− 0.048	0.69
SLICC/ACR-DI	0.14	0.26
SLEDAI-2 K	− 0.048	0.69
VAS pain	0.27	0.021
MADRS-S	0.11	0.35
VAS fatigue	0.31	0.008
Fatigue Severity Scale	0.18	0.12

r_s_: Spearman’s rank correlation coefficient. SLICC: Systemic Lupus Erythematosus International Collaborating Clinics. ACR: American College of Rheumatology. DI: Damage Index. SLEDAI-2 K: SLE Disease Activity Index 2000. VAS: Visual Analogue Scale 100 mm. MADRS-S: Montgomery-Åsberg Depression Rating Scale – Self-rated version

Serum S100A8/A9 did not correlate with age (r_s_ = 0.054, *p* = 0.65), disease duration (r_s_ = − 0.048, *p* = 0.069), SLEDAI (r_s_ = − 0.048, *p* = 0.069), SLICC/ACR-DI (r_s_ = 0.14, *p* = 0.26), the extent of white matter lesions (r_s_ = 0.02, *p* = 0.88), brain volumes or with neurocognitive test scores (CNS-VS) (data not shown). CSF S100A8/A9 concentrations did not correlate with serum S100A8/A9 (r_s_ = 0.019, *p* = 0.92), or with the abovementioned variables (data not shown). Increased CSF albumin quotient was present in 3 of 33 patients, and oligoclonal bands specific for CSF were present in 8 of 33 patients, of whom 3 had strong bands. No significant associations were observed in the abovementioned analyses when excluding patients with increased CSF albumin quotient in the models (data not shown).

### Multivariate analysis of NPSLE

A multivariate analysis investigating the association between NPSLE and serum S100A8/A9 corrected for confounders was only performed for the ACR model due to the small group sizes of the SLICC A and B models. In this model serum S100A8/A9, scores of FSS and VAS pain were included as covariates. In this analysis, NPSLE was not significantly associated with higher serum S100A8/A9 concentrations when adjusting for VAS pain and FSS (OR 1.86, 95% CI 0.93–3.73, *p* = 0.08). MADRS-S and VAS fatigue were not included in the multivariate analysis due to multicollinearity. The depression scores were not independent of fatigue scores, and as expected VAS fatigue scores were strongly correlated with Fatigue Severity Scale as well as VAS pain scores. Furthermore, depression may be an NPSLE manifestation in itself, and depression scores (MADRS-S) were strongly correlated with NPSLE.

## Discussion

Although the exact role and underlying mechanisms of S100A8/A9 in CNS pathology remain unclear, increasing evidence has demonstrated that S100A8/A9 is closely related to certain CNS diseases [12, 18–25]. S100A8/A9 has also been associated with SLE, and serum S100A8/A9 concentrations has been shown to be correlated with disease activity and decrease after immunosuppressive treatment [13–17]. In this cross-sectional study we found an association between higher S100A8/A9 concentrations in serum and neuropsychiatric involvement in SLE, which was not seen when analysing anti-dsDNA or complement factor 3 and 4. S100A8/A9 was detectable in the CSF of SLE patients. Nevertheless, we could not find an association between CSF levels of S100A8/A9 and NPSLE. One possible explanation may be the difficulty of determining NPSLE, thus leading to misclassification, but also the probability that several pathogenetic mechanisms are operating in NPSLE, which would influence our findings. Patients with ongoing NP symptoms may not have active neuroinflammation, but would still be included in the NPSLE patient group. A study design assessing patients with new-onset NPSLE symptoms could possibly ameliorate this issue. Correspondingly, subclinical neuroinflammation may be present in non-NPSLE-patients. This may particularly be a confounding factor when applying the more stringent SLICC A and B application models for NPSLE. We did not have CSF samples from the healthy control group to compare with, and no reference to illustrate if the levels are high or low other than a study measuring S100A8/A9 in the CSF at concentrations around 100 pg/ml in healthy controls and 250 pg/ml in AD patients [19], which was lower than the median concentrations in this study.

### S100A8/A9 in NPSLE immunopathogenesis

The possible role of S100A8/A9 in NPSLE immunopathogenesis remains unclear. Constantly, the immunologically privileged milieu in the CNS receives messages from the peripheral immune system, and cells of the CNS can become immunologically active upon stimulation. The subsequent cross-talking between microglia, oligodendrocytes, astrocytes and neurons can result in re-modulation or degeneration in “classical” neuroimmune diseases such as MS, but even in neurological and psychiatric disorders, which historically have not been associated with neuroinflammation such as depression, AD, Parkinson’s disease and stroke [35]. In support of a pathogenetic role of S100A8/A9 in neuroinflammation, *Wu *et al. demonstrated that local treatment with S100A8/A9 in murine brains induced the activation, proliferation and migration of microglial cells, and that the treated microglial cells in vitro switched from an anti-inflammatory activated phenotype to a pro-inflammatory activated phenotype, inducing the release of pro-inflammatory factors and chemokines via the NFκB-pathway, ultimately causing degeneration of brain tissue [26]. *Gong *et al. demonstrated that S100A8/A9 was upregulated in both the hippocampus and serum of mice after exposure to chronic stress which resulted in depressive behaviours. In addition, central administration of S100A8/A9 resulted in neuroinflammation via TLR4/NFκB-signaling leading to depressive behaviours, and pharmacological intervention with TLR4-inhibitors resulted in an attenuation of these effects [23]. In our study, SLE patients with depression as an NPSLE manifestation according to the ACR model had higher S100A8/A9 concentrations in serum compared with non-NPSLE patients, supporting their suggestion of a pathogenetic contribution.

S100A8/A9 in the CNS may be a result of either local production from cells in the CNS or from systemically produced S100A8/A9 crossing the blood–brain barrier, possibly contributing to neuropsychiatric manifestations, although we could not confirm this hypothesis in this study. Neutrophils are the main source of S100A8/A9 in the circulation with concentrations 1000 times higher than CSF [10]. The possibility that blood neutrophils from the lumbar puncture could affect CSF concentrations cannot be excluded. In MS, a disease that exclusively affects the CNS, patients had significantly higher serum levels of S100A8/A9 than healthy controls (medians 5150 ng/ml and 1482 ng/ml respectively), and MS patients with acute relapse had higher levels than MS patients with stable disease [18].

The elevated serum S100A8/A9 concentrations seen in our SLE patients with neuropsychiatric involvement may solely represent systemic SLE-related inflammation. Our study was cross-sectional and patients were consecutively asked to participate independently of neuropsychiatric symptoms or SLE activity. NPSLE symptoms were not new onset and the majority of patients were on immunosuppressive treatment. The higher serum S100A8/A9 levels in the NPSLE patients may indicate an ongoing SLE-related systemic inflammatory process, not completely controlled by immunosuppression, possibly contributing to neuropsychiatric symptoms. This is mirrored by the finding of an interferon signature in clinically inactive SLE patients [36]. Hypothetically, a potential indirect mechanism of S100A8/A9 in NPSLE is through its action on endothelial cells contributing to vasculopathy and atherosclerosis development [37]. Serum S100A8/A9 concentrations did not correlate with disease activity assessed with SLEDAI-2 K, a finding that has been demonstrated in two studies [13, 15]. In our prevalent SLE patients, SLEDAI-2 K was generally low, which would decrease the chance of detecting an association with disease activity. We also compared serum S100A8/A9 levels in SLE patients with healthy controls, and although our control group was small, the results were in line with previous studies, demonstrating significantly higher serum S100A8/A9 concentrations in SLE patients [14–16].

S100A8/A9 has previously been investigated in childhood-onset SLE with neurocognitive disorders. There was no significant difference of serum S100A8/A9 concentrations in these patients with or without neurocognitive disorders (mean 1540 ng/ml and 929 ng/ml respectively, *p* = 0.25), however, only 9 and 31 patients respectively were included in this study [38]. In the same study a significant correlation between serum S100A8/A9 levels and cognitive disability over time was demonstrated, which is consistent with our finding that patients with the NPSLE manifestation “cognitive dysfunction” according to the ACR model had higher serum levels of S100A8/A9 compared with non-NPSLE patients, although the degree of cognitive dysfunction in the majority of patients was mild. Longitudinal studies are needed to investigate the role of S100A8/A9 in the development of cognitive dysfunction in SLE.

### S100A8/A9 and fatigue

The aetiology of fatigue in SLE patients is likely multi-factorial, including medication, affective disorders, chronic pain, cognitive dysfunction and organ damage, albeit emerging evidence suggests that during chronic inflammation, various pathophysiological mechanisms negatively affect brain functioning in areas that are involved in fatigue by increasing glutaminergic and decreasing monoaminergic neurotransmission [7, 9, 39]. Previous studies have demonstrated that depression, stress, anxiety and pain are independently associated with fatigue in SLE [40, 41]. The majority of our SLE patients had clinically significant fatigue and the frequencies are consistent with previous SLE studies [32, 41, 42]. Our results illustrate that fatigue often persists even after achieving low disease activity, which is observed in other chronic inflammatory diseases [7]. Fatigue and pain scores were also positively correlated with higher serum S100A8/A9 levels, although the associations were not strong. Fatigue may be part of subtle SLE activity systemically or in the CNS, although further studies are needed to investigate the association between S100A8/A9 and fatigue, and the mechanisms involved. In our multivariate analysis serum S100A8/A9 was not independently associated with NPSLE when correcting for pain and fatigue scores. One possible explanation is that the included variables may not be unrelated to each other rendering estimates from a multivariate analysis unreliable. It may be difficult for a patient to assess which percentage of their fatigue or pain is caused by the disease itself, and consequently, the reliability of the subjective VAS measurements may be questioned. Furthermore, the size of the study precludes a firm conclusion from multivariate models.

Our study has limitations. The study is cross-sectional, which makes it impossible to determine causes and effects. The small sizes of the studied groups result in difficulties drawing firm conclusions and our findings need to be confirmed in larger studies, especially for individual NP manifestations. The methodology of solely including female patients with a certain age threshold increases the chance of finding significant associations, particularly since the NPSLE syndrome is heterogenous, however, the usefulness of the results is limited to that group. The study did not include disease controls with primary psychiatric or neurological disease, and therefore we were not able to draw conclusions on the discriminatory abilities of S100A8/A9 from other conditions. CSF was not obtained from the controls, and less than half of SLE patients consented to lumbar puncture. Most patients were in remission or had generally low clinically discernible activity in their SLE, thus the results are only applicable in this context. The associations between S100A8/A9 and the ACR model should be regarded with caution, due to the ACR model being suggested to overestimate NPSLE [5, 28]. However, the careful clinical patient evaluation by two specialists in the study determining SLE attribution would reduce this concern. An overestimation of NPSLE would influence results towards not finding significant associations. The patients were included consecutively, however, patients with NP symptoms may to a higher degree have accepted to participate in this study. Consequently, the study group is not strictly unselected and this possible participation bias may have resulted in higher frequencies of NPSLE manifestations than could be expected in a random SLE population. The psychiatric manifestations were based on questionnaires and clinical assessments by a neurologist and rheumatologist rather than by a psychiatrist which may result in imprecise prevalences. Although these factors may contribute to a less accurate estimation of the frequency of NPSLE, this would not affect the possible associations with S100A8/A9. The study has strengths too. Considering the low prevalence of NPSLE, and the relatively small amount of literature on this topic, the aspects of NP-symptoms in SLE patients were described in detail involving a specialist in neurology, CSF and blood analysis, MRI assessments, and cognitive testing by a neuropsychologist.

## Conclusions

In conclusion, NPSLE may be associated with higher serum S100A8/A9 concentrations. Higher serum S100A8/A9 may be associated with a higher degree of fatigue. The results suggest that S100A8/A9 may be of interest in neuropsychiatric involvement of SLE, although further investigations are warranted to determine the usefulness in clinical practice, in particular longitudinal studies.

## Data Availability

The authors confirm that the data supporting the conclusions of this study are available within the article.

## Abbreviations

SLE: Systemic lupus erythematosus
NPSLE: Neuropsychiatric systemic lupus erythematosus
CNS: Central nervous system
TLR4: Toll-like receptor 4
RAGE: Receptor of advanced glycation endproducts
MS: Multiple sclerosis
CSF: Cerebrospinal fluid
AD: Alzheimer’s disease
ACR: American College of Rheumatology
SLICC: Systemic lupus erythematosus international collaborating clinics
DI: Damage index
SLEDAI-2 K: SLE disease activity index 2000
FSS: Fatigue severity scale
VAS: Visual analogue scale 100 mm
MADRS-S: Montgomery-Åsberg Depression Rating Scale – Self-rated version
CNS VS: CNS vital signs
DMARD: Disease-modifying anti-rheumatic drug
ANA: Antinuclear Antibody
Anti-dsDNA: Anti-double stranded DNA
Anti-Sm: Anti-smith
Anti-PL: Anti-phospholipid antibody
